# Prognostic Roles of Glucose to Lymphocyte Ratio and Modified Glasgow Prognosis Score in Patients With Non-small Cell Lung Cancer

**DOI:** 10.3389/fnut.2022.871301

**Published:** 2022-05-10

**Authors:** Ming Yang, Qi Zhang, Yi-Zhong Ge, Meng Tang, Chun-Lei Hu, Zi-Wen Wang, Xi Zhang, Meng-Meng Song, Guo-Tian Ruan, Xiao-Wei Zhang, Tong Liu, Hai-Lun Xie, He-Yang Zhang, Kang-Ping Zhang, Qin-Qin Li, Xiang-Rui Li, Xiao-Yue Liu, Shi-Qi Lin, Han-Ping Shi

**Affiliations:** ^1^Department of Gastrointestinal Surgery / Department of Clinical Nutrition, Beijing Shijitan Hospital, Capital Medical University, Beijing, China; ^2^Beijing International Science and Technology Cooperation Base for Cancer Metabolism and Nutrition, Beijing, China; ^3^Key Laboratory of Cancer FSMP for State Market Regulation, Beijing, China; ^4^The Second Affiliated Hospital & Yuying Children's Hospital of Wenzhou Medical University, Wenzhou, China

**Keywords:** glucose to lymphocyte ratio, modified Glasgow Prognosis Score, non-small cell lung cancer, inflammation, nutrition, immune

## Abstract

**Background:**

Non-small cell lung cancer (NSCLC) is among the most prevalent malignancies worldwide. Previous studies have shown that the status of inflammation, nutrition and immune are closely related to overall survival (OS) of patients with NSCLC, but little is known about their interactive and combined roles. Hence, we chose glucose to lymphocyte ratio (GLR) and modified Glasgow Prognosis Score (mGPS) as prognostic factors and assessed the prognostic values of them for patients with NSCLC.

**Methods:**

Baseline clinicopathologic and laboratory characteristics of 862 patients with NSCLC were obtained from a multicenter prospective cohort. The Cox proportional hazard regression models were used to determine prognostic values of the clinical factors. A nomogram was also constructed integrating the clinical factors with clinical significance or independent prognostic values. Concordance index (C-index) was utilized to evaluate the prediction accuracy of the TNM stage and the nomogram.

**Results:**

Multivariate analyses demonstrated that GLR [Hazard ratio (HR) = 1.029, 95% confidence interval (CI) = 1.004–1.056, *P* = 0.023] and mGPS (score of 1: HR = 1.404, 95% CI = 1.143–1.726, *P* = 0.001; score of 2: HR = 1.515, 95% CI = 1.159–1.980, *P* = 0.002) were independent prognostic factors for patients with NSCLC. The C-indexes of the TNM stage and the nomogram were 0.642 (95% CI = 0.620–0.663) and 0.694 (95% CI = 0.671–0.717), respectively.

**Conclusion:**

GLR and mGPS were independent prognostic factors for patients with NSCLC. Moreover, our constructed nomogram might be superior in predicting prognosis of patients with NSCLC compared with the TNM stage.

## Introduction

Non-small cell lung cancer (NSCLC), which is a common type of lung cancer, is the leading cause of cancer-related death worldwide, bringing a tremendous burden to families and society (1, 2). In previous studies, numerous prognostic factors were identified to better predict survival and inform clinical decisions for patients with NSCLC (3–10). Due to limitations of these studies, however, existing prognostic factors are inadequate to meet the growing needs for better prediction of survival and informing more effective treatment strategies for patients with NSCLC (11, 12). Therefore, development of better prediction models would result in better therapy decisions and would be beneficial to improve outcomes for patients with NSCLC.

Previous studies have shown that inflammation is an important promoting factor for the occurrence and development of lung cancer (13). The risk of death in patients with NSCLC with high levels of inflammation is much higher than those with low levels of inflammation. Elevated fasting blood glucose (FBG) is not only the direct embodiment of insulin resistance caused by inflammation, but also the direct cause of further inflammation. C-reactive protein (CRP), as a common clinical index, is very sensitive to the changes of inflammatory level.

On the other hand, the nutritional and immune status of patients with NSCLC are also crucial to their survival. Some studies suggested that the survival of patients with NSCLC, with poor nutritional and lymphocyte status, is worse than those with good status, and this gap can be corrected by nutritional supplement and immune intervention (14, 15). In addition to being an immune marker, recent studies have reported that lymphocytes are closely related to the nutritional status of the body (16). Moreover, serum albumin (Alb) has been used as a nutritional index in clinic for a long time.

Therefore, we identified glucose to lymphocyte ratio (GLR), which is a parameter that integrates both FBG levels and lymphocyte counts, and the modified Glasgow prognostic score (mGPS), which combines Alb and CRP, to be prognostic factors with high accuracy in patients with NSCLC. GLR and mGPS are previously reported prognostic indicators of a variety of cancers. We reasoned that a combination of both GLR and mGPS would be more promising in prediction of OS for patients with NSCLC. Thus, we established a nomogram model that combined GLR and mGPS. We showed that this nomogram was more accurate and specific in predicting prognosis for patients with NSCLC.

The current study aimed to evaluate the prognostic values of GLR, mGPS and a nomogram model that combined GLR and mGPS in patients with NSCLC.

## Materials and Methods

### Study Population

**Figure 1:**
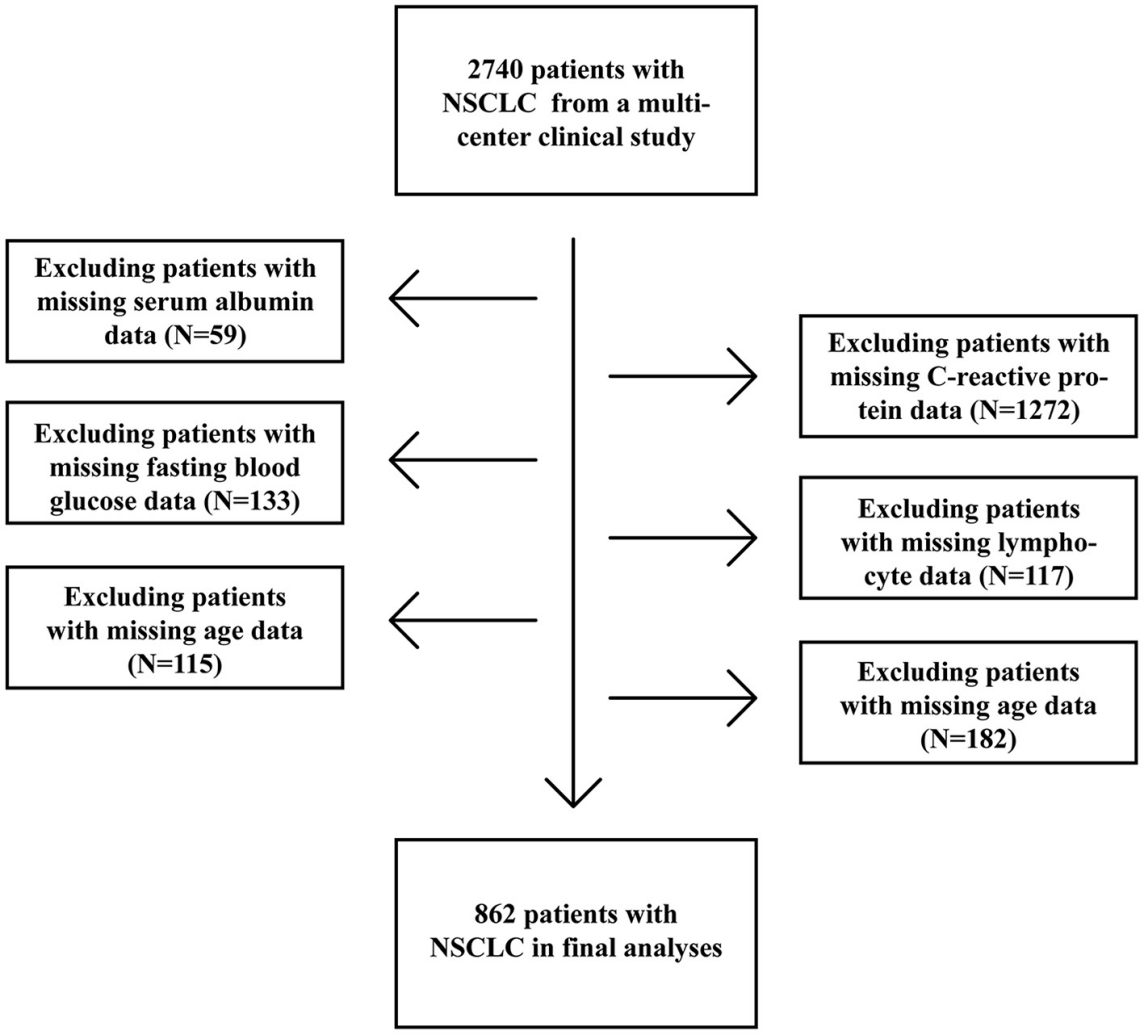
Procedures for selection of study participants with non-small cell lung cancer (NSCLC) from a multi-center clinical database.

A total of 2,740 patients with NSCLC, who were diagnosed between 2012 and 2019, were enrolled from a multicenter prospective cohort which included patients from 14 hospitals (Figure 1). The inclusion criteria were as following: age ≥ 18 years at diagnosis, a pathological diagnosis of NSCLC, willing and able to provide written informed consent, and consciousness throughout treatment with no communication disorders. Patients with any of the following conditions will be excluded: acquired immune deficiency syndrome, mental or cognitive impairment, or receiving organ transplantation. Cases which patients were hospitalized for more than 2 times during the study were considered as single cases. 1,878 patients without reported data for one or more of above variables were excluded. Of these excluded patients, 59 were missing Alb levels, 1,272 were missing CRP levels, 133 were missing FBG levels, 117 were missing lymphocyte counts, 115 were missing age information, and 182 were missing TNM stage data (Figure 1). The study was approved by the Ethical Review Committees of the participating hospitals and was conducted in accordance with the guidelines of the Declaration of Helsinki. The study was registered with the Chinese Clinical Trial Registry (http://www.chictr.org.cn) and the registration number is ChiCTR1800020329.

### Patient Characteristics and Outcomes

The following demographic and clinicopathological data were collected within 24 h of admission: sex, age, height, weight, smoking status, alcohol consumption, tea-drinking status, health information (hypertension, diabetes and coronary heart disease), TNM stage, FBG levels, lymphocyte counts, Alb and CRP levels. Smoking more than 20 cigarettes in a lifetime was regarded as smoking. Regular alcohol consumption in the past year was regarded as drinking. Regular tea drinking in the past year was regarded as tea drinking. TNM stages were determined according to the guidelines of the American Joint Committee on Cancer (17). Blood samples were taken at 6 a.m. the next day after the patient's admission. Before that, patients were required to be fasting for at least 8 h. The mGPS was defined as following: arbitrary Alb levels and CRP ≤ 10 mg/L were scored as 0; Alb ≥ 35 g/L or CRP > 10 mg/L were scored as 1; Alb <35 g/L and CRP > 10 mg/L were scored as 2. GLR was the level of FBG divided by the lymphocyte counts. Patient death due to NSCLC was defined as the primary endpoint of the clinical trials.

### Statistical Analyses

Data were presented as simple percentages or medians and interquartile range (IQR). The Fisher's exact test or chi-square test was used for the assessment of baseline characteristics. Student's *t* test was utilized for the analyses of continuous variables with normal distributions. The Mann-Whitney test was used for the analyses of continuous variables with non-normal distributions.

The prognostic values of different variables were evaluated by the univariate and multivariate Cox proportional hazard regression models. Our proposed nomogram model was constructed based on these identified prognostic factors. Concordance index (C-index) and Area Under the Curve (AUC) were used to evaluate the accuracy of our nomogram model. The Cox regression models were used for hazard ratio (HR) and associated 95% confidence interval (CI). *P* < 0.05 was regarded as statistically significant. For all the analyses, either the R (version 4.0.1) or SPSS (version 26.0) software was employed.

## Results

### Patient Characteristics

Of all the participants, the median age was 61 years (IQR, 54 to 67 years), the median BMI was 22.99 kg/m^2^ (IQR, 20.81 to 25.10 kg/m^2^), the median GLR was 3.66 (IQR, 2.76 to 5.18). 64.2% (553/862) of the patients were male. 8.0% (69/862), 15.2% (131/862), 23.5% (203/862) and 53.2% (459/862) of them were in stage I, II, III, and IV, respectively. According to the values of mGPS, the baseline characteristics were divided into three groups and were summarized in Table 1.

**Table 1:** Characteristics of patients with different mGPS.

**Characteristics**	**mGPS = 0 (*n* = 507)**	**mGPS = 1 (*n* = 254)**	**mGPS = 2 (*n* = 101)**	***P*-value**
Sex*^a^* (male)	300 (59.2)	177 (69.7)	76 (75.2)	0.001
Age in years*^b^*	59.71 (9.29)	61.22 (9.77)	62.99 (10.76)	0.003
BMI*^b^* (kg/m^2^)	23.38 (3.31)	22.91 (3.15)	21.95 (3.25)	<0.001
Smoking status*^ac^* (Yes)	282 (55.6)	161 (63.4)	69 (68.3)	0.018
Alcohol consumption*^a,d^* (Yes)	128 (25.2)	70 (27.6)	31 (30.7)	0.481
Tea drinking status*^a,e^* (Yes)	107 (21.1)	68 (26.8)	33 (32.7)	0.023
Hypertension*^a^* (Yes)	91 (17.9)	67 (26.4)	24 (23.8)	0.021
Diabetes*^a^* (Yes)	43 (8.5)	26 (10.2)	7 (6.9)	0.561
Coronary heart disease*^a^* (Yes)	29 (5.7)	20 (7.9)	8 (7.9)	0.452
**TNM stage** *^***a***^*				<0.001
I	56 (11.0)	10 (3.9)	3 ( 3.0)	
II	88 (17.4)	33 (13.0)	10 ( 9.9)	
III	126 (24.9)	58 (22.8)	19 (18.8)	
IV	237 (46.7)	153 (60.2)	69 (68.3)	
GLR*^b^*	4.28 (2.91)	4.89 (3.16)	4.84 (3.10)	0.018

*mGPS, modified Glasgow prognostic score; BMI, body mass index; GLR, blood glucose to lymphocyte ratio*.

a *Categorical variables are presented as number (percentage)*.

b *Continuous variables are presented as mean (standard deviation)*.

c *The standard is to smoke more than 20 cigarettes in a lifetime*.

d *The standard is regular drinking in the past year*.

e *The standard is regular drinking tea in the past year*.

### Independent Prognostic Factors for NSCLC

Univariate analyses indicated that sex, BMI, tea drinking status, TNM stage, mGPS and GLR were prognostic factors for patients with NSCLC, while age, smoking status, alcohol consumption, and health status (hypertension, diabetes and coronary heart disease) were not. Multivariate analyses further indicated that sex (HR = 0.814, 95% CI = 0.666–0.995, *P* = 0.023), BMI (HR = 0.939, 95% CI = 0.913–0.966, *P* < 0.001), TNM stage (stage II: HR = 3.718, 95% CI = 1.762–7.847, *P* = 0.001; stage III: HR = 6.466, 95% CI = 3.153–13.258, *P* < 0.001; stage IV: HR = 10.205, 95% CI = 5.048–20.632, *P* < 0.001), mGPS (score of 1: HR = 1.404, 95% CI = 1.143-1.726, *P* = 0.001; score of 2: HR = 1.515, 95% CI = 1.159-1.980, *P* = 0.002) and GLR (HR = 1.029, 95% CI = 1.004–1.056, *P* = 0.023) were independent prognostic factors. The detailed results of these analyses were summarized in Table 2.

**Table 2:** Associations between clinical variables and OS in patients with NSCLC.

**Variables**	**Univariate analysis**		**Multivariate analysis*^a^***	
	**HR (95% CIs)**	***P*-value**	**HR (95% CIs)**	***P*-value**
Sex	0.789 (0.655, 0.951)	0.013	0.814 (0.666, 0.995)	0.023
Age	1.009 (0.999, 1.018)	0.066	1.000 (0.990, 1.009)	0.972
BMI	0.927 (0.906, 0.953)	<0.001	0.939 (0.913, 0.966)	<0.001
Smoking status*^b^*	1.127 (0.941, 1.349)	0.193		
Alcohol consumption*^c^*	0.954 (0.781, 1.164)	0.640		
Tea drinking status*^d^*	1.236 (1.013, 1.508)	0.037	1.104 (0.892, 1.367)	0.365
Hypertension	1.064 (0.859, 1.319)	0.568		
Diabetes	0.956 (0.699, 1.306)	0.775		
Coronary heart disease	0.965 (0.674, 1.381)	0.845		
**TNM stage**				
I	Reference		Reference	
II	3.824 (1.813, 8.066)	<0.001	3.718 (1.762, 7.847)	0.001
III	7.064 (3.449, 14.469)	<0.001	6.466 (3.153, 13.258)	<0.001
IV	11.548 (5.721, 23.310)	<0.001	10.205 (5.048, 20.632)	<0.001
**mGPS**				
0	Reference		Reference	
1	1.879 (1.550, 2.277)	<0.001	1.404 (1.143, 1.726)	0.001
2	2.174 (1.669, 2.831)	<0.001	1.515 (1.159, 1.980)	0.002
GLR	1.038 (1.013, 1.063)	0.002	1.029 (1.004, 1.056)	0.023

*OS, overall survival; NSCLC, non-small cell lung cancer; HR, hazard ratio; CIs, confidence interval; BMI, body mass index; mGPS, modified Glasgow prognostic score; GLR, blood glucose to lymphocyte ratio*.

a *The variables showed prognosis roles in univariate analysis or considered clinically significant were involved in multivariate analysis*.

b *The standard is to smoke more than 20 cigarettes in a lifetime*.

c *The standard is regular drinking in the past year*.

d *The standard is regular drinking tea in the past year*.

### GLR and mGPS Prognostic Roles

Correlation analyses indicated that the risk of death was positively related to GLR levels (Table 3, Supplementary Figure 1). Receiver operating characteristic curve (ROC) analyses determined that the optimal cut-off value for GLR was 4.726. Patients with a GLR > 4.726 had a lower OS compared with patients who had a GLR smaller than or equal to 4.726 (HR = 1.501, 95% CI = 1.246–1.808, *P* < 0.001; Supplementary Figures 2, 3). When GLR was divided into 4 quartiles (1^st^ quartile: GLR <2.760; 2^nd^ quartile: 2.760 ≤ GLR <3.662; 3^rd^ quartile: 3.662 ≤ GLR <5.194; 4^th^ quartile: GLR ≥ 5.194), patients in the 4^th^ quartile had a significantly higher risk of death (HR = 1.662, 95% CI = 1.292–2.138, *P* < 0.001) compared to those in the 1^st^ quartile. For mGPS, patients with a score of 1 or 2 had a significantly decreased survival time compared to those with a score of 0. Detailed associations between mGPS and OS in patients with NSCLC were presented in Table 3, Supplementary Figure 4.

**Table 3:** Associations between GLR or mGPS and OS in patients with NSCLC.

**Variables**	**Patients (*n*)**	**Crude HR (95% CIs)**	***P*-value**	**Adjusted HR (95% CIs)**	***P*-value**
**GLR** *^***a***^*					
Continuous	862	1.038 (1.013, 1.063)	0.002	1.029 (1.004, 1.056)	0.023
**Categories** *^***b***^*					
Low	604	Reference		Reference	
High	258	1.691 (1.408, 2.030)	<0.001	1.501 (1.246, 1.808)	<0.001
**Quartiles**					
1	215	Reference		Reference	
2	216	1.147 (0.884, 1.487)	0.302	1.122 (0.864, 1.457)	0.388
3	216	1.249 (0.966, 1.615)	0.089	1.185 (0.914, 1.536)	0.199
4	215	1.877 (1.468, 2.400)	<0.001	1.662 (1.292, 2.138)	<0.001
**mGPS** *^***c***^*					
0	507	Reference		Reference	
1	254	1.723 (1.406, 2.111)	<0.001	1.404 (1.143, 1.726)	0.001
2	101	2.021 (1.559, 2.621)	<0.001	1.515 (1.159, 1.980)	0.002
**GLR and mGPS** *^***d***^*					
Low GLR and 0 score	382	Reference		Reference	
High GLR and 0 score	125	1.790 (1.371, 2.336)	<0.001	1.564 (1.220, 2.006)	<0.001
Low GLR and 1 score	157	2.024 (1.590, 2.577)	<0.001	1.499 (1.153, 1.948)	0.002
High GLR and 1 score	97	2.477 (1.878, 3.267)	<0.001	1.910 (1.413, 2.583)	<0.001
Low GLR and 2 score	65	2.077 (1.482, 2.912)	<0.001	1.425 (1.014, 2.003)	0.042
High GLR and 2 score	36	3.686 (2.485, 5.467)	<0.001	2.554 (1.705, 3.824)	<0.001

*GLR, blood glucose to lymphocyte ratio; mGPS, modified Glasgow prognostic score; OS, overall survival; NSCLC, non-small cell lung cancer; HR, hazard ratio; CIs, confidence intervals*.

a *Models were adjusted for sex, age, body mass index, tea drinking status, TNM stage and mGPS*.

b *The cut-off point of GLR is 4.726*.

c *Model was adjusted for sex, age, body mass index, tea drinking status, TNM stage and GLR (as a continuous variable)*.

d *Model was adjusted for sex, age, body mass index, tea drinking status and TNM stage*.

### Combination Prognostic Roles

Based on different combinations of GLR and mGPS, all patients were assigned into six groups: group 1 (Low GLR and mGPS = 0), group 2 (High GLR and mGPS = 0), group 3 (Low GLR and mGPS = 1), group 4 (High GLR and mGPS = 1), group 5 (Low GLR and mGPS = 2), and group 6 (High GLR and mGPS = 2). Compared with patients in group 1, lower OS was observed for patients in group 2 (HR = 1.564, 95% CI = 1.220–2.006, *P* < 0.001), group 3 (HR = 1.499, 95% CI = 1.153–1.948, *P* = 0.002), group 4 (HR = 1.910, 95% CI = 1.413–2.583, *P* < 0.001), group 5 (HR = 1.425, 95% CI = 1.014–2.003, *P* = 0.042), and group 6 (HR = 2.554, 95% CI = 1.705–3.824, *P* < 0.001). Detailed results were summarized in Table 2.

### Evaluation of the Constructed Nomogram

**Figure 2:**
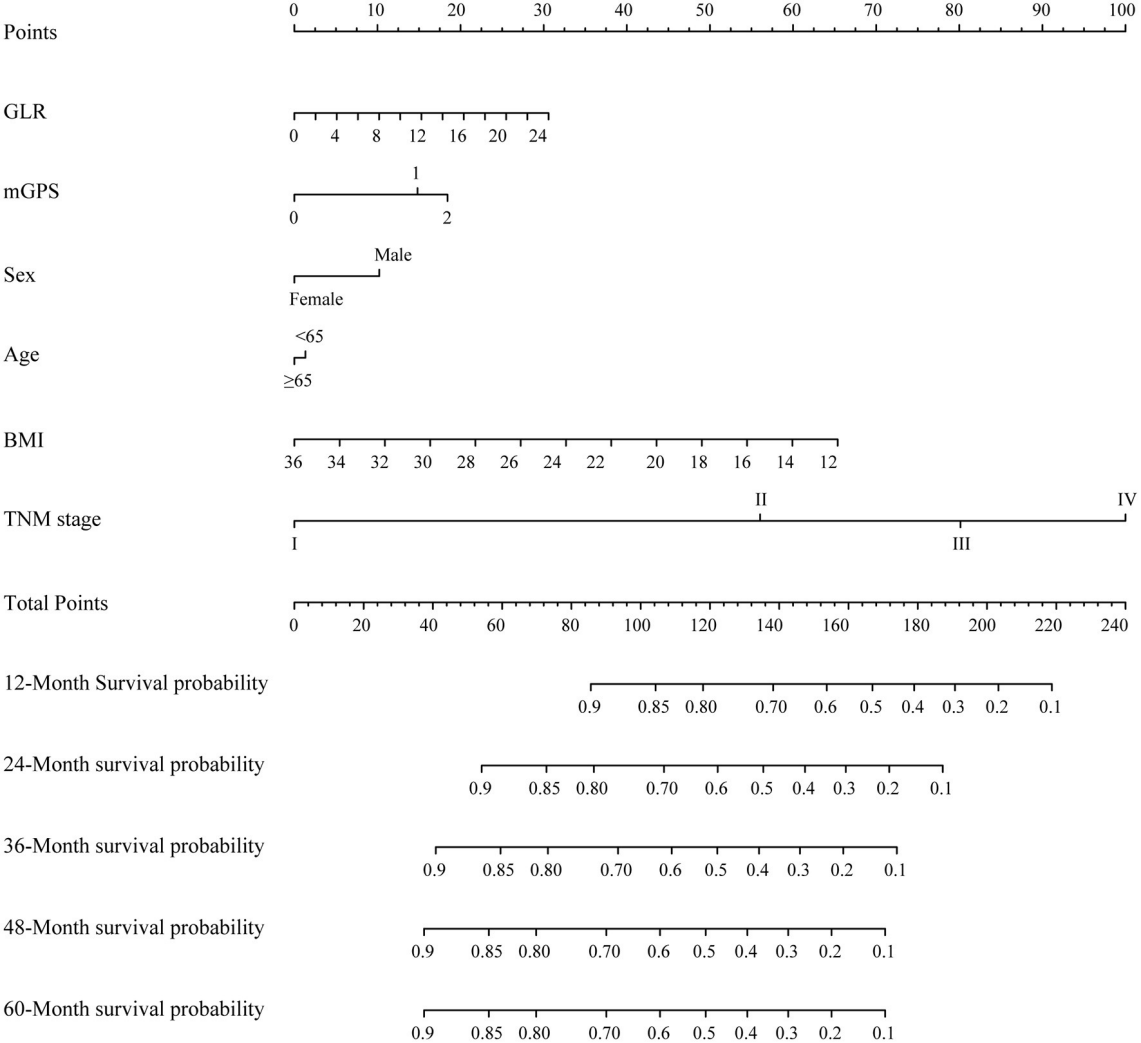
A proposed nomogram for predicting median survival time and survival probability of patients with non-small cell lung cancer (NSCLC). To use the nomogram, a line is drawn upward to the Points axis to determine the number of points received for each variable. Sum of these points makes the total points. For total points, a line is drawn from the Total Points axis downward to the survival axes to determine the estimated median survival time and survival probability.

Variables, with clinical significance (age) or with independent prognostic value (TNM stage, BMI, GLR, mGPS, sex), were included in the constructed nomogram (Figure 2). The calibration curves for 1-, 2- and 3-year OS were in good agreement with prediction from our nomogram (Supplementary Figure 5). C-indexes of the TNM stage and the nomogram were 0.642 (95% CI, 0.620–0.663) and 0.694 (95% CI, 0.671–0.717), respectively. Time-dependent ROCs were generated based on the GLR, mGPS, TNM stage and our nomogram. AUCs of ROCs generated from the TNM stage and our nomogram were 68.48 and 74.54% at 1 year, 67.74 and 73.27% at 2 years, 73.16 and 76.82% at 3 years, and 77.59 and 81.69% at 4 years, respectively (Supplementary Figure 6). These data suggested that our nomogram model might performs better in predicting OS compared with the classical TNM stage classification in patients with NSCLC (Supplementary Figure 7).

## Discussion

In the current study, we concluded that GLR and mGPS were negatively correlated with OS in patients with NSCLC. We also confirmed that clinical factors, such as sex, BMI and TNM stage, were independent risk factors for patients with NSCLC. Further, we developed a nomogram model that incorporated NSCLC prognostic factors, which might provide more accurate prediction of OS than the TNM staging system or other traditional indicators included in the nomogram in patients with NSCLC. Using c-indexes and AUC, we showed that our nomogram model was superior in predicting outcomes of patients with NSCLC compared to the classical TNM stage classification method.

GLR, which is the ratio of FBG levels and lymphocyte counts, was shown to be a good predictor of OS in several malignancies, such as pancreatic and gallbladder tumors (18, 19). Level of FBG was also found to be independent predictor for OS in patients with NSCLC (20). Diabetes can lead to hyperinsulinemia and insulin resistance, which can promote tumor growth (21, 22). Moreover, previous studies showed that hyperglycemia, which could form irreversible glycation end-products, could change the tumor microenvironment and contribute to tumor development (23). Previous studies also demonstrated that circulating lymphocytes can contribute to improved outcomes in cancer patients by enhancing cancer immune-surveillance (13, 24). In the tumor microenvironment, lack of T cells indicated impaired anti-tumor immunity (25). More than that, lymphocytes, which are important cells in immunity, also indicate patient's nutritional status (16). Research indicated that nutrition education and oral supplementation can both improve nutritional status as well as the activity of lymphocytes, which will improve the prognosis of patients with NSCLC (14, 15, 26). In conclusion, the elevated GLR, representing hyperglycemia and low lymphocyte count, is closely related to cancer progression and poor prognosis, which is consistent with our results. Interestingly, regulatory T cells were activated under low glucose levels, countering anti-tumor immunity (27–29). Thus, the potential synergism between hypoglycemia and immunosuppression might block cancer progression. Further studies could be carried out to verify this hypothesis and explore the possible mechanisms.

It was reported that mGPS, combining Alb levels and CRP levels, was identified as a prognostic marker for patients with NSCLC (30). As an inflammatory index, decreased Alb levels indicated malnutrition and systemic inflammation (31). Studies had shown that malnutrition and systemic inflammation are closely related to the adverse outcomes of patients with NSCLC (32). Moreover, increased CRP levels were linked to lymphocytopenia and impaired T cell response in tumors, which can further promote cancer progression (33). It is recognized that ongoing systemic inflammatory responses (indicated by an elevated CRP) in cancer patients can lead to poor survival due to reasons such as increased protein breakdown (34–37). Hence, higher mGPS, meaning hypoalbuminemia and elevated CRP levels, is associated with a poor prognosis in patients with NSCLC, which is consistent with our conclusion.

In this study, we explored how a combination of GLR and mGPS would relate to OS of patients with NSCLC. A higher death risk was found in patients with high GLR and mGPS = 2, compared with those who have a low GLR and mGPS = 0. Higher GLR and mGPS mean higher FBG and CRP levels, and lower lymphocyte count and albumin levels, which are associated with high inflammatory status, malnutrition and immune insufficiency. Studies had shown that systemic inflammation, malnutrition and immune dysfunction are related to the progression of cancer, which will lead to worse survival (38, 39). More than that, these indicators and states will also affect each other. Prior studies identified an association between inflammatory status and the occurrence of diabetes. Inflammatory responses likely contribute to diabetes occurrence by regulating insulin resistance, which in turn intensifies the symptoms of hyperglycemia and further promotes long-term complications of diabetes (40). Moreover, by activating macrophages, hypoalbuminemia can impair the immune response within tumors (41). Hence, we reasoned that integration of systemic inflammatory state, malnutrition and inhibition of immune function could provide a more comprehensive and accurate prediction of OS, compared to any of those single factors.

In conclusion, lowering FBG levels, activating immune system, improving systemic inflammation levels, and maintaining adequate nutrition could improve the prognosis of patients with NSCLC. It should be noted that while parenteral and enteral nutrition was an important way of nutritional support for patients with NSCLC, it could also potentially lead to hyperglycemia (42). Therefore, it would be advisable to maintain an appropriate FBG levels while actively carry out nutritional interventions (43).

In addition to GLR and mGPS, our model also included sex, age, BMI and TNM stage. Previous studies had shown that age, TNM stage and BMI are predictors for OS of patients with NSCLC (44–46). In this study, we showed that our constructed nomogram might be superior in predicting OS of patients with NSCLC compared with any of above factors included in the nomogram, providing a basis for personalized treatment and clinical applications.

**Figure 3:**
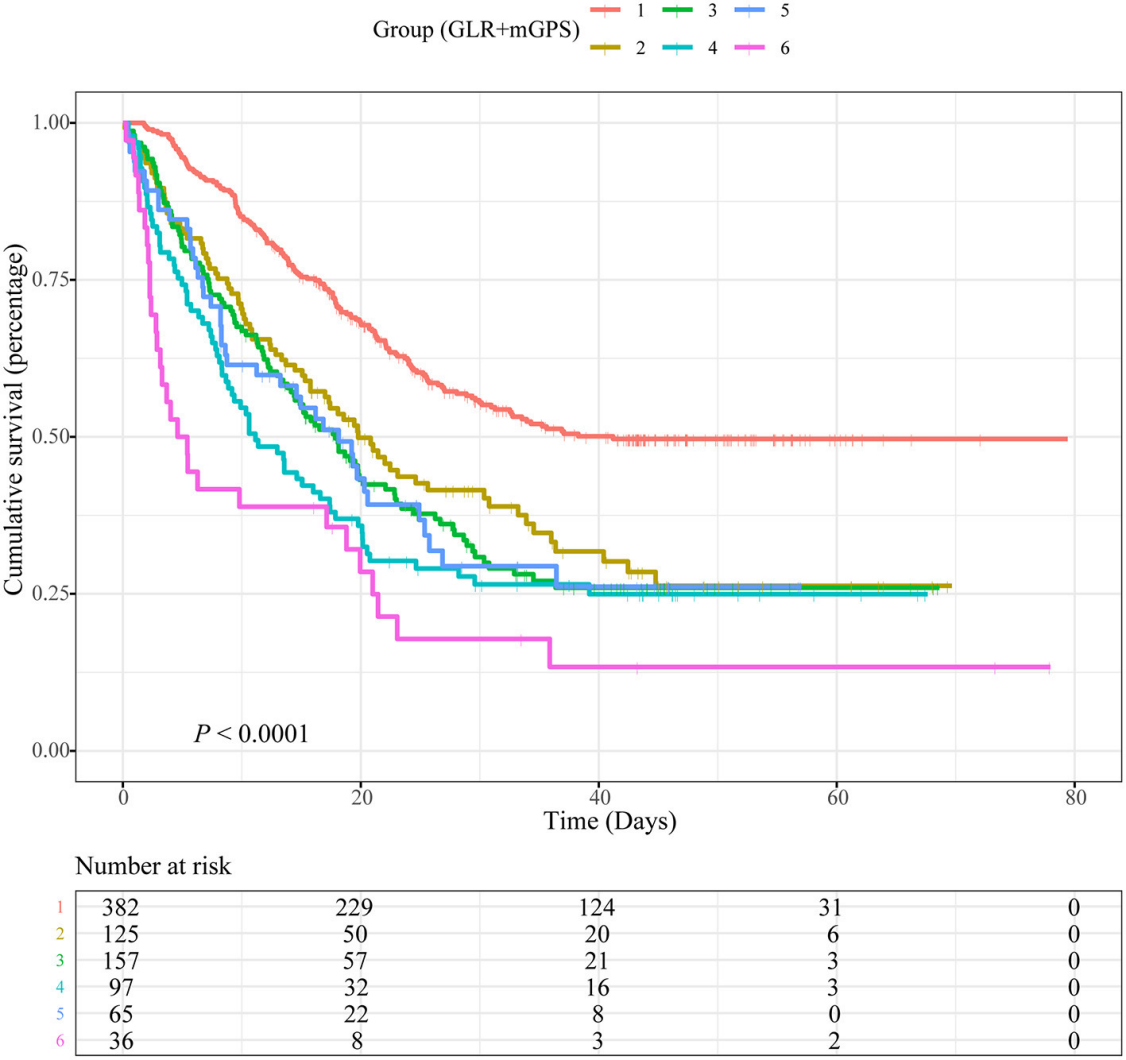
Kaplan-Meier curves showing associations between a combination of blood glucose to lymphocyte ratio (GLR) and modified Glasgow prognostic score (mGPS) and overall survival (OS) in patients with non-small cell lung cancer (NSCLC). Group 1: Low GLR and mGPS = 0; Group 2: High GLR and mGPS = 0; Group 3: Low GLR and mGPS = 1; Group 4: High GLR and mGPS = 1; Group 5: Low GLR and mGPS = 2; Group 6: High GLR and mGPS = 2.

However, our study might have flaws in several ways. The sample sizes were relatively small, which might affect the statistical power. It is worth mentioning that in Figure 3, when GLR is higher than 6.25, the association between GLR and risk of death for patients with NSCLC was no longer significant. Based on threshold analyses, piecewise regression analyses and population distribution analyses, we suspected that this was due to the small sample size (Supplementary Figure 8, Supplementary Table 1). In addition, laboratory data were determined using standard laboratory tests, which were limited in scope compared to more advanced testing techniques. Studies with larger sample size and more clinical factors should be carried out in the future to further improve OS prediction for patients with NSCLC. Because the records of NSCLC treatment in our database were not detailed enough, such as the interval between admission and operation, chemotherapy or radiotherapy, the operation mode of operation, the scheme and specific dose of chemotherapy, the radiation dose of radiotherapy, etc., we believed that adding these treatment data to the multivariate analysis might affect the reliability and stability of the results. Therefore, we did not adjust for the treatments. In future research, we will improve the deficiencies mentioned above in the database and record the treatment data of patients in more detail.

## Conclusion

In summary, GLR and mGPS could be used as independent prognostic factors for survival of patients with NSCLC. The proposed nomogram could predict OS of patients with NSCLC with good sensitivity and specificity. Compared to the TNM staging system or other traditional indicators included in the nomogram, our proposed nomogram might provide a more accurate and specific tool for facilitating clinical decisions, personalized treatment, and disease management in patients with NSCLC.

## Data Availability Statement

The raw data supporting the conclusions of this article will be made available by the authors, without undue reservation.

## Ethics Statement

The studies involving human participants were reviewed and approved by the Ethical Review Committees of the participating hospitals. The patients/participants provided their written informed consent to participate in this study.

## Author Contributions

H-PS: conceptualization and methodology. MY: data curation and writing- original draft preparation. QZ: visualization, investigation, and data curation. Y-ZG, TL, and K-PZ: software. MT: validation and visualization. C-LH, XZ, M-MS, H-LX, and X-YL: writing-reviewing and editing. Z-WW, G-TR, X-WZ, H-YZ, Q-QL, X-RL, and S-QL: supervision and investigation. All authors contributed to the article and approved the submitted version.

## Funding

This work was supported by the National Key Research and Development Program (2017YFC1309200).

## Conflict of Interest

The authors declare that the research was conducted in the absence of any commercial or financial relationships that could be construed as a potential conflict of interest.

## Publisher's Note

All claims expressed in this article are solely those of the authors and do not necessarily represent those of their affiliated organizations, or those of the publisher, the editors and the reviewers. Any product that may be evaluated in this article, or claim that may be made by its manufacturer, is not guaranteed or endorsed by the publisher.

## Supplementary Material

Supplementary Figure 1 Associations between blood glucose to lymphocyte ratio (GLR) and overall survival (OS) in patients with non-small cell lung cancer (NSCLC). Model was adjusted for sex, age, BMI, tea drinking status, TNM stage and modified Glasgow prognostic score (mGPS).

Supplementary Figure 2 Receiver operating characteristic curve (ROC) for determining the cut-off point of blood glucose to lymphocyte ratio (GLR).

Supplementary Figure 3 Kaplan-Meier curves showing associations between blood glucose to lymphocyte ratio (GLR) and overall survival (OS) in patients with non-small cell lung cancer (NSCLC).

Supplementary Figure 4 Kaplan-Meier curves showing associations between modified Glasgow prognostic score (mGPS) and overall survival (OS) in patients with non-small cell lung cancer (NSCLC).

Supplementary Figure 5 Calibration curves for predicting survival probability of patients with non-small cell lung cancer (NSCLC).

Supplementary Figure 6 Area Under the Curves (AUCs) of time-dependent receiver operating characteristic curves (ROCs) generated based on the TNM stage and the nomogram for patients with non-small cell lung cancer (NSCLC).

Supplementary Figure 7 Area Under the Curves (AUCs) of TNM stage and the nomogram in patients with non-small cell lung cancer (NSCLC).

Supplementary Figure 8 Associations between blood glucose to lymphocyte ratio (GLR <6.25 or ≥ 6.25) and OS in patients with non-small cell lung cancer (NSCLC). The models were adjusted for sex, age, BMI, tea drinking status, TNM stage and modified Glasgow prognostic score (mGPS).

Supplementary Table 1 Associations between GLR and OS in patients with NSCLC. GLR, blood glucose to lymphocyte ratio; OS, overall survival; NSCLC, non-small cell lung cancer; HR, hazard ratio; CIs, confidence intervals. a Models were adjusted for sex, age, body mass index, tea drinking status, TNM stage and mGPS.
