# Supplementary figures and images for: Prognostic Roles of Glucose to Lymphocyte Ratio and Modified Glasgow Prognosis Score in Patients With Non-small Cell Lung Cancer
Source: Front Nutr. 2022 May 10;9:871301. doi: 10.3389/fnut.2022.871301 (PMC9127733; doi:10.3389/fnut.2022.871301)

**(A)**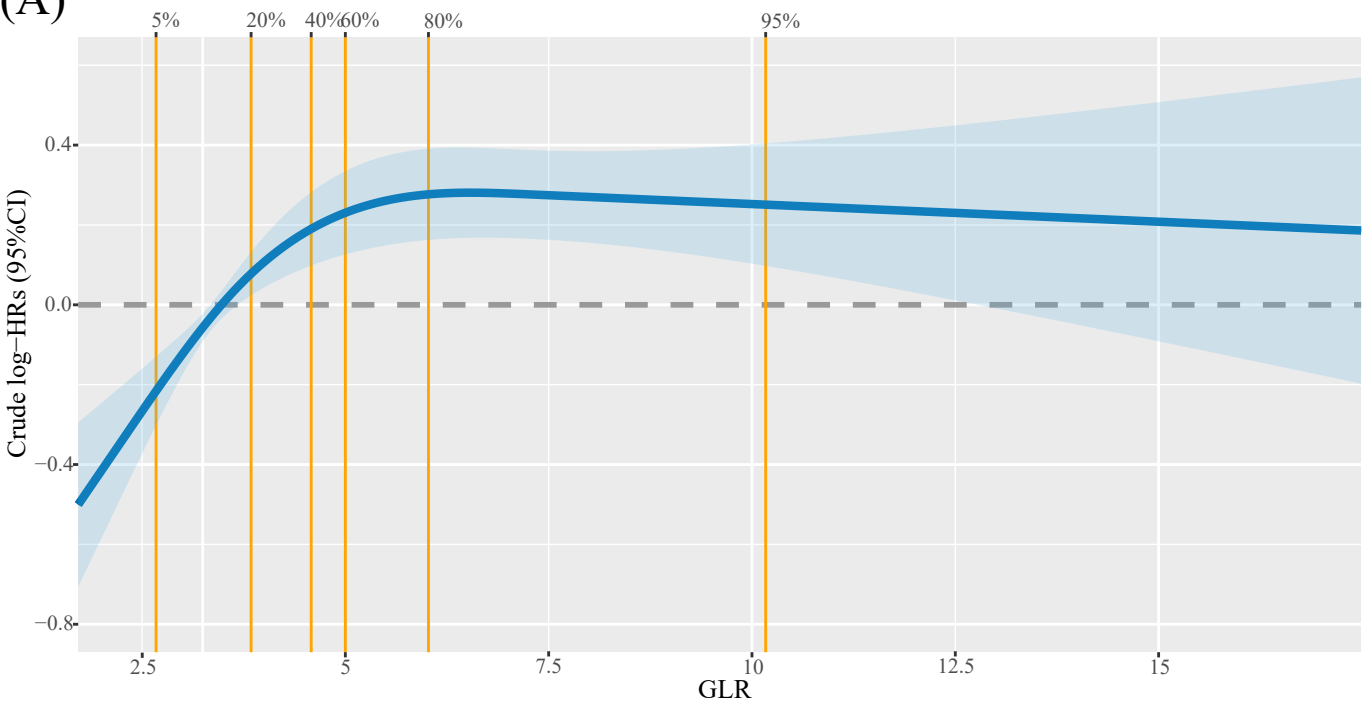**(B)**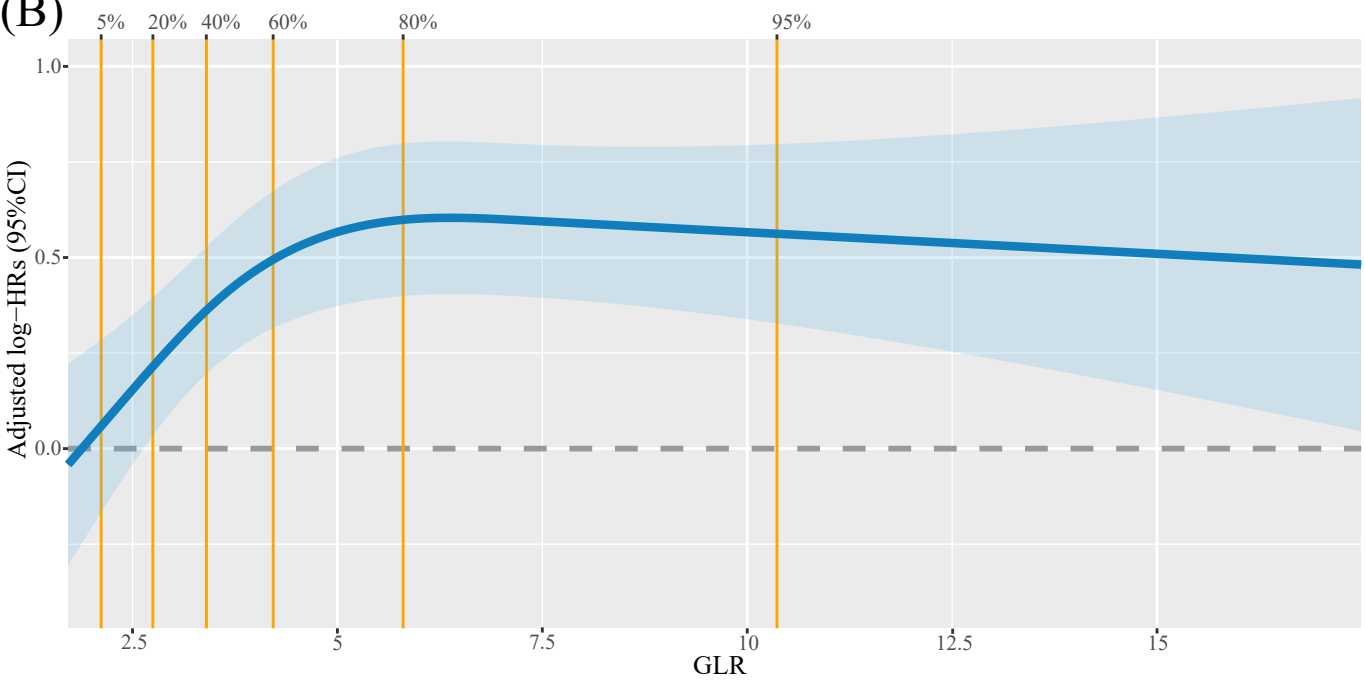

Supplement: Supplementary Figure 1 — Associations between blood glucose to lymphocyte ratio (GLR) and overall survival (OS) in patients with non-small cell lung cancer (NSCLC). Model was adjusted for sex, age, BMI, tea drinking status, TNM stage and modified Glasgow prognostic score (mGPS). [file Image_1.PDF]

# GLR

## Distribution

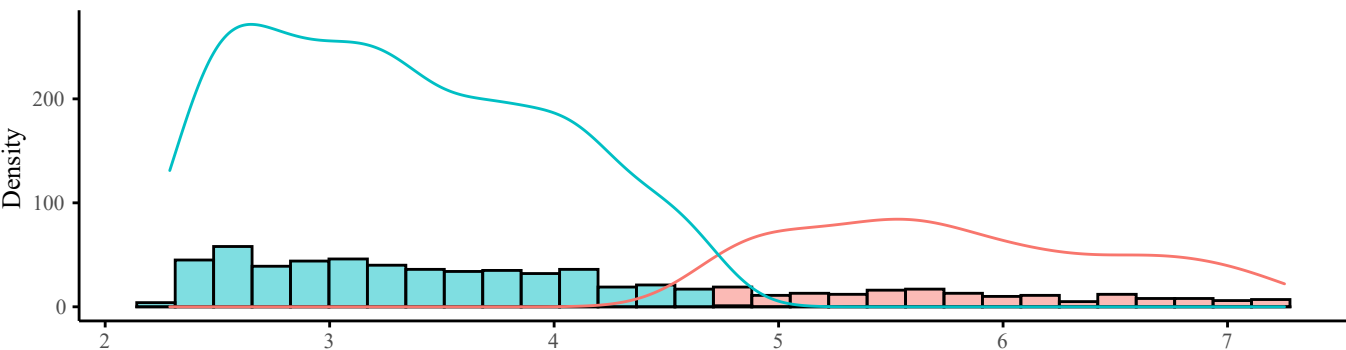

## Maximally Selected Rank Statistics

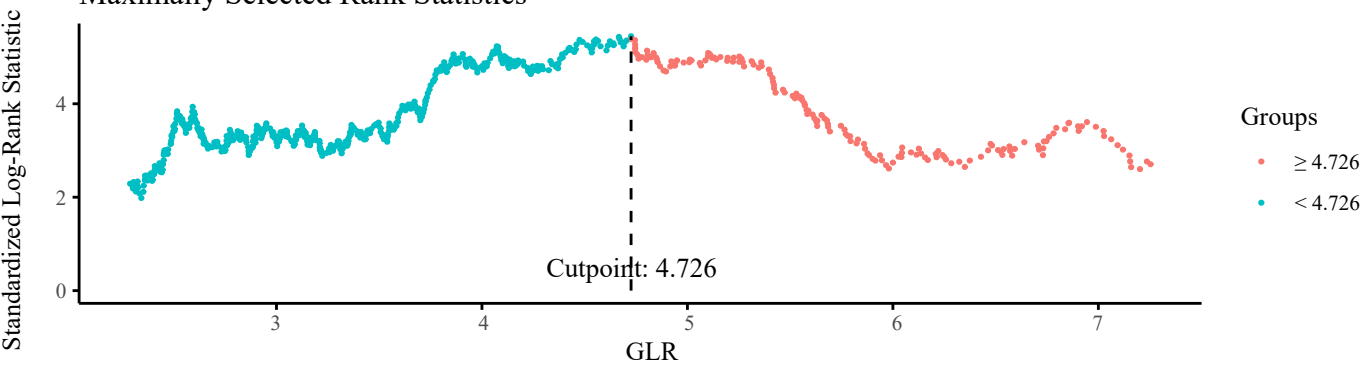

Supplement: Supplementary Figure 2 — Receiver operating characteristic curve (ROC) for determining the cut-off point of blood glucose to lymphocyte ratio (GLR). [file Image_2.PDF]

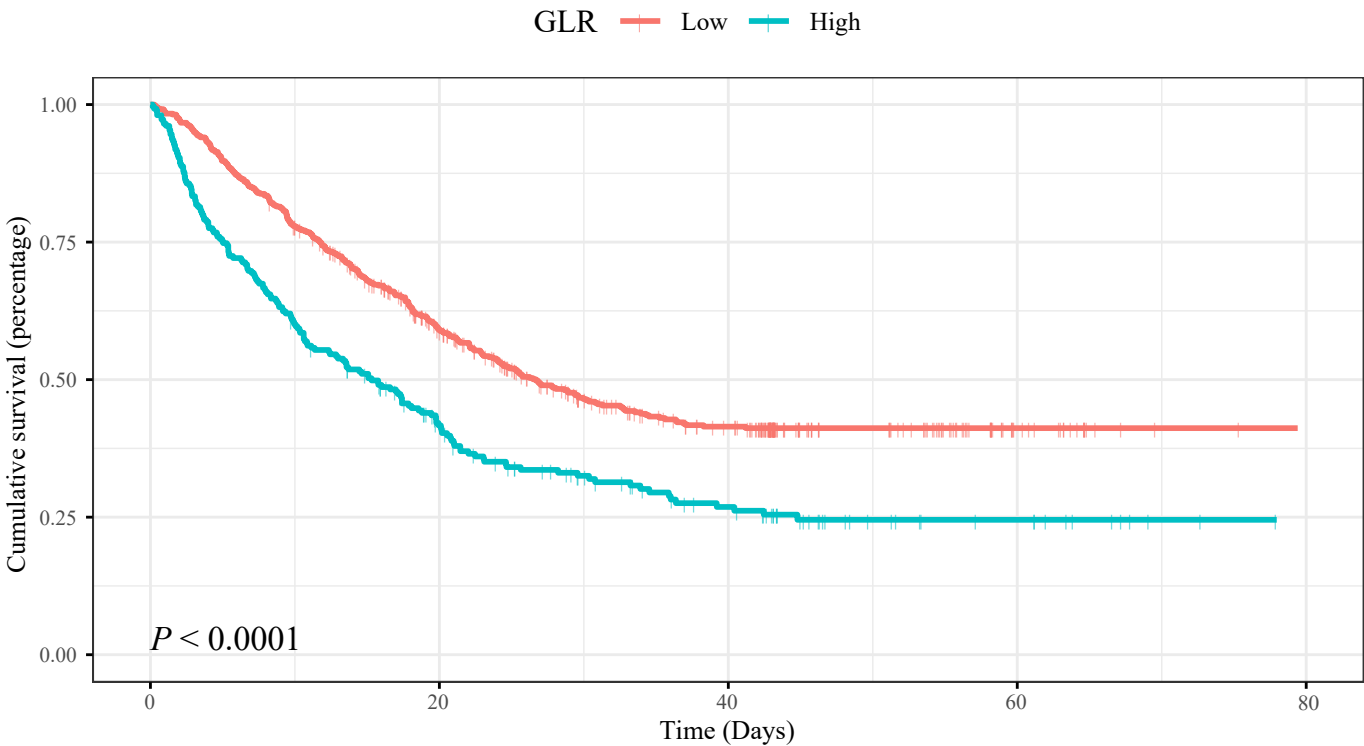

Number at risk

Low

604

308

153

34

0

High

258

90

39

11

0

Supplement: Supplementary Figure 3 — Kaplan-Meier curves showing associations between blood glucose to lymphocyte ratio (GLR) and overall survival (OS) in patients with non-small cell lung cancer (NSCLC). [file Image_3.PDF]

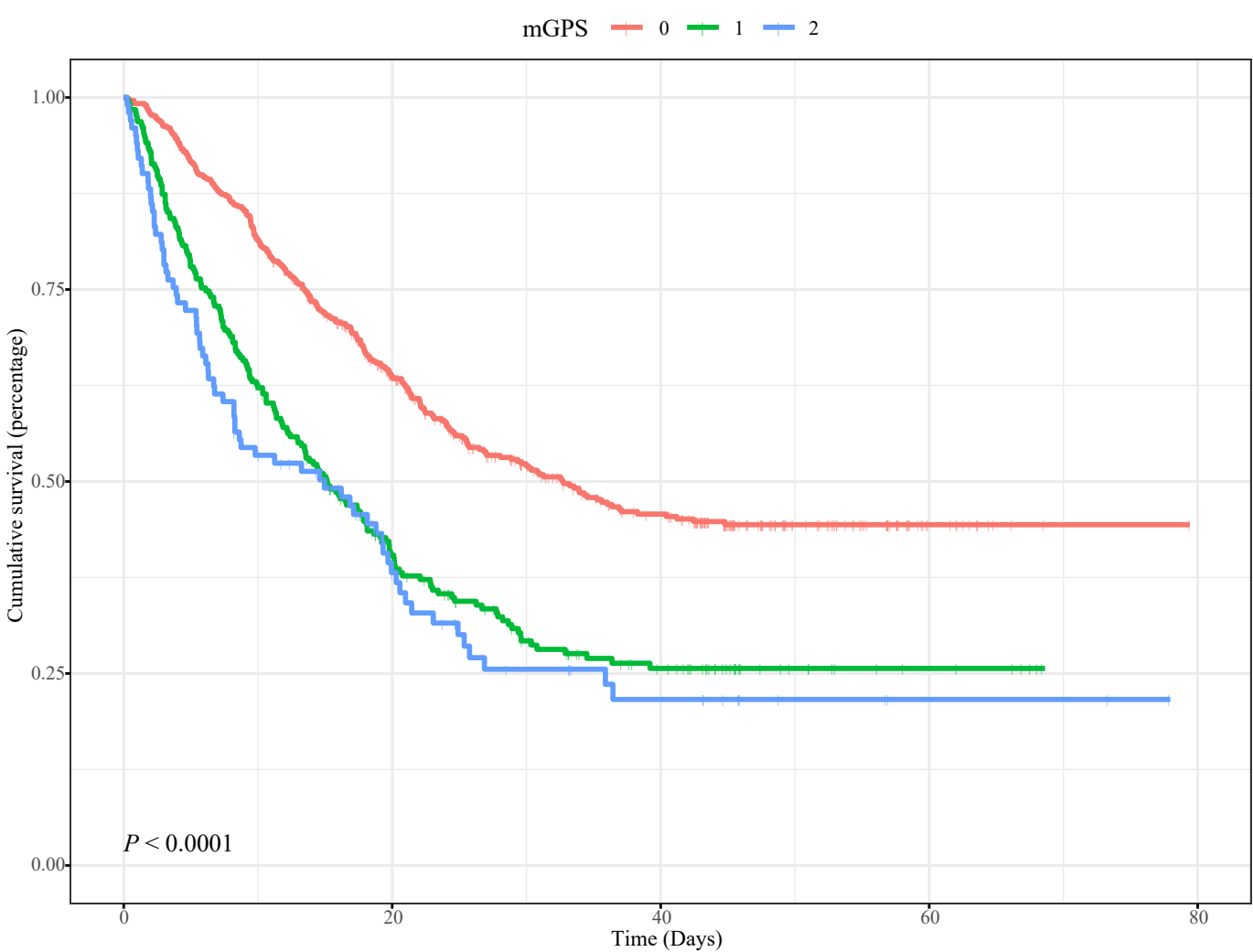

Number at risk

|   |     |     |     |    |   |
|---|-----|-----|-----|----|---|
| 0 | 507 | 279 | 144 | 37 | 0 |
| 1 | 254 | 89  | 37  | 6  | 0 |
| 2 | 101 | 30  | 11  | 2  | 0 |

Supplement: Supplementary Figure 4 — Kaplan-Meier curves showing associations between modified Glasgow prognostic score (mGPS) and overall survival (OS) in patients with non-small cell lung cancer (NSCLC). [file Image_4.PDF]

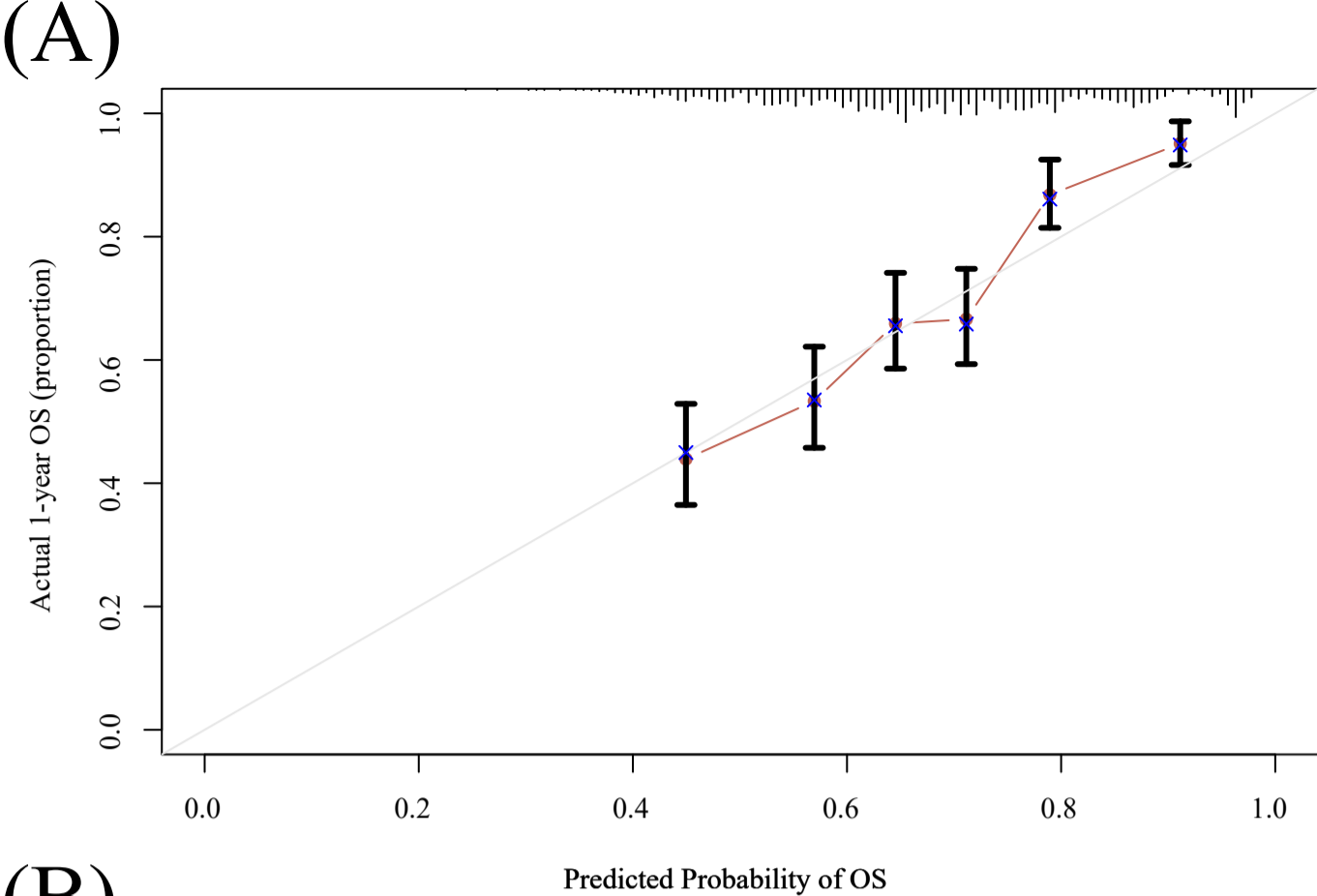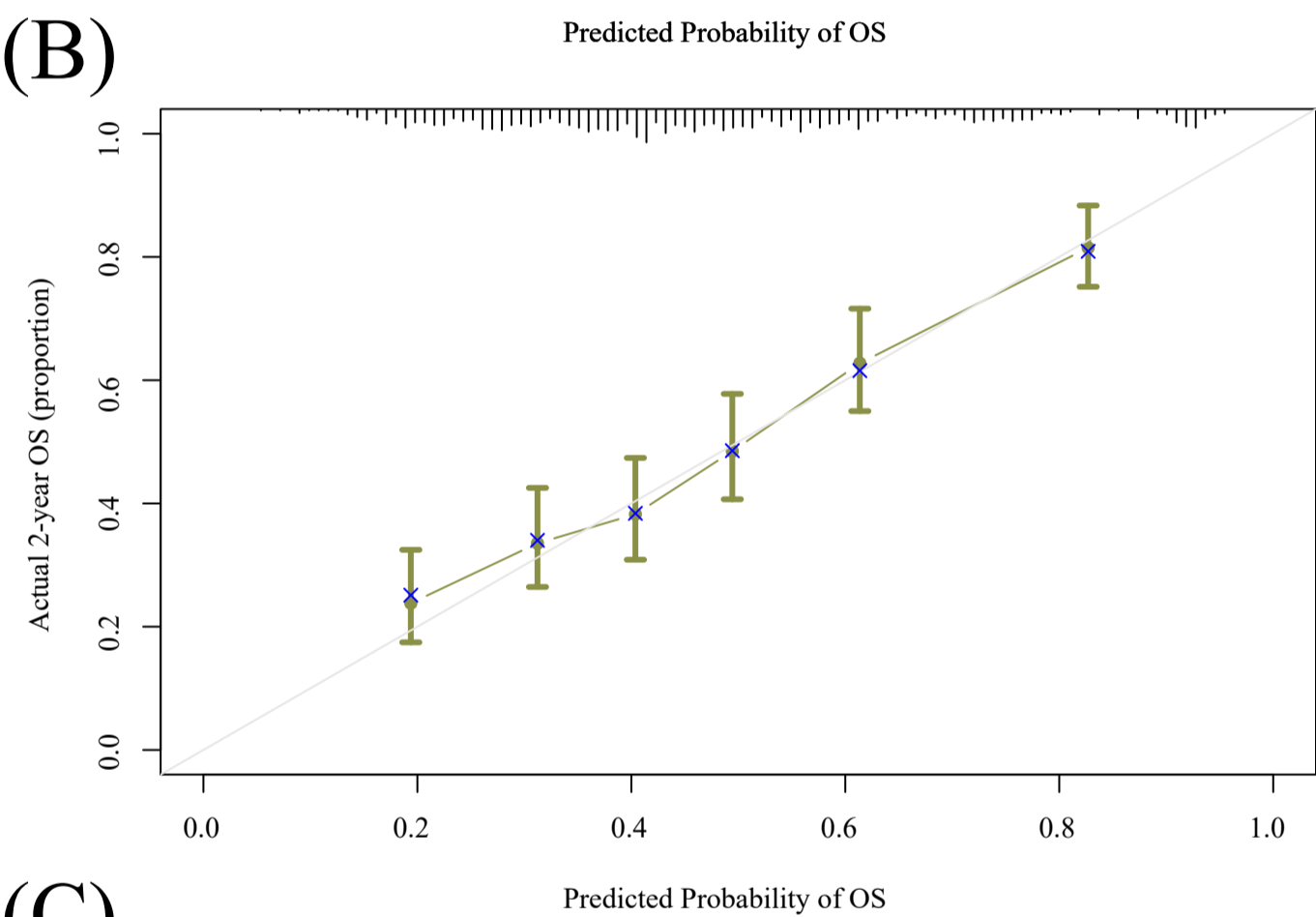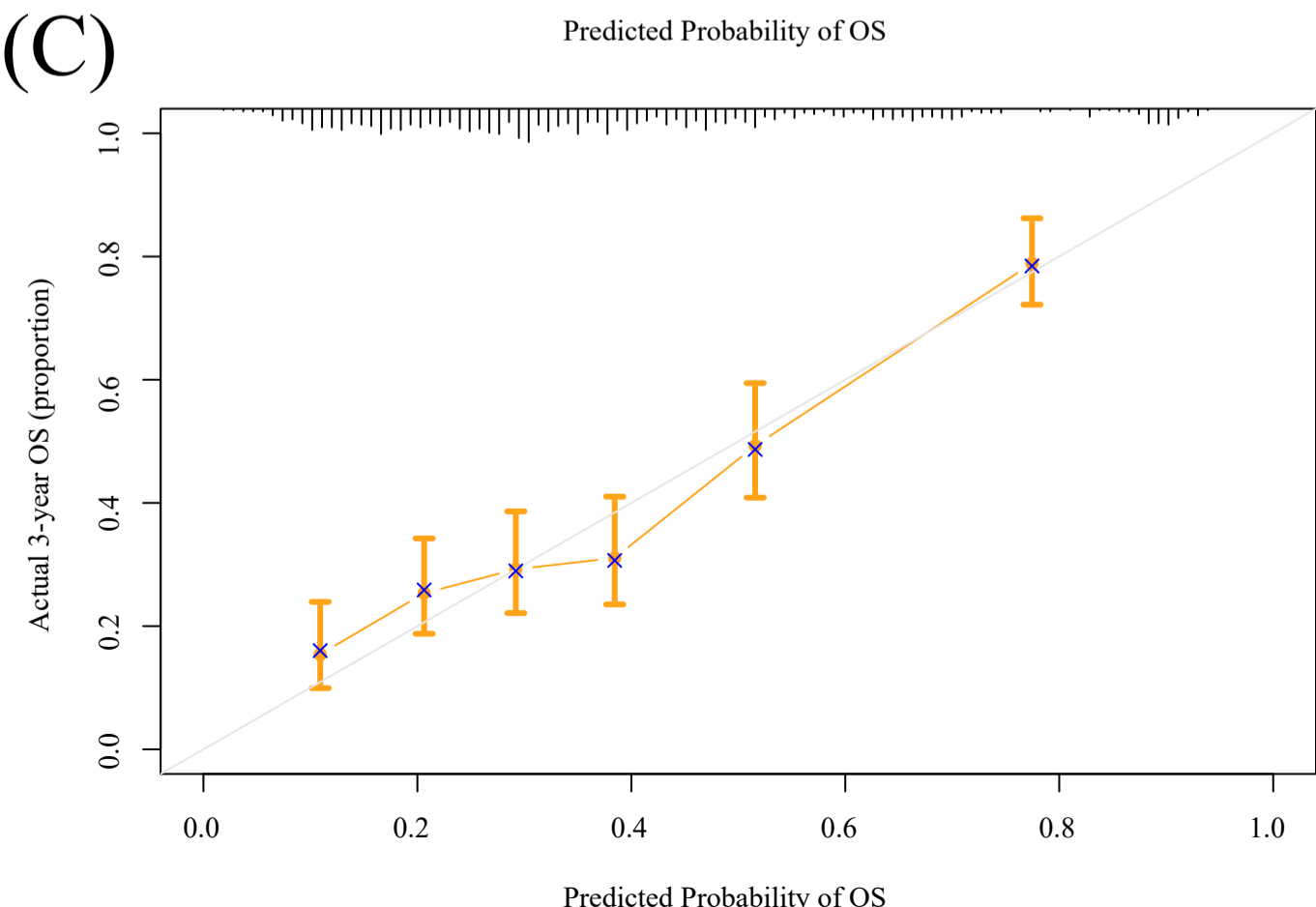

Supplement: Supplementary Figure 5 — Calibration curves for predicting survival probability of patients with non-small cell lung cancer (NSCLC). [file Image_5.PDF]

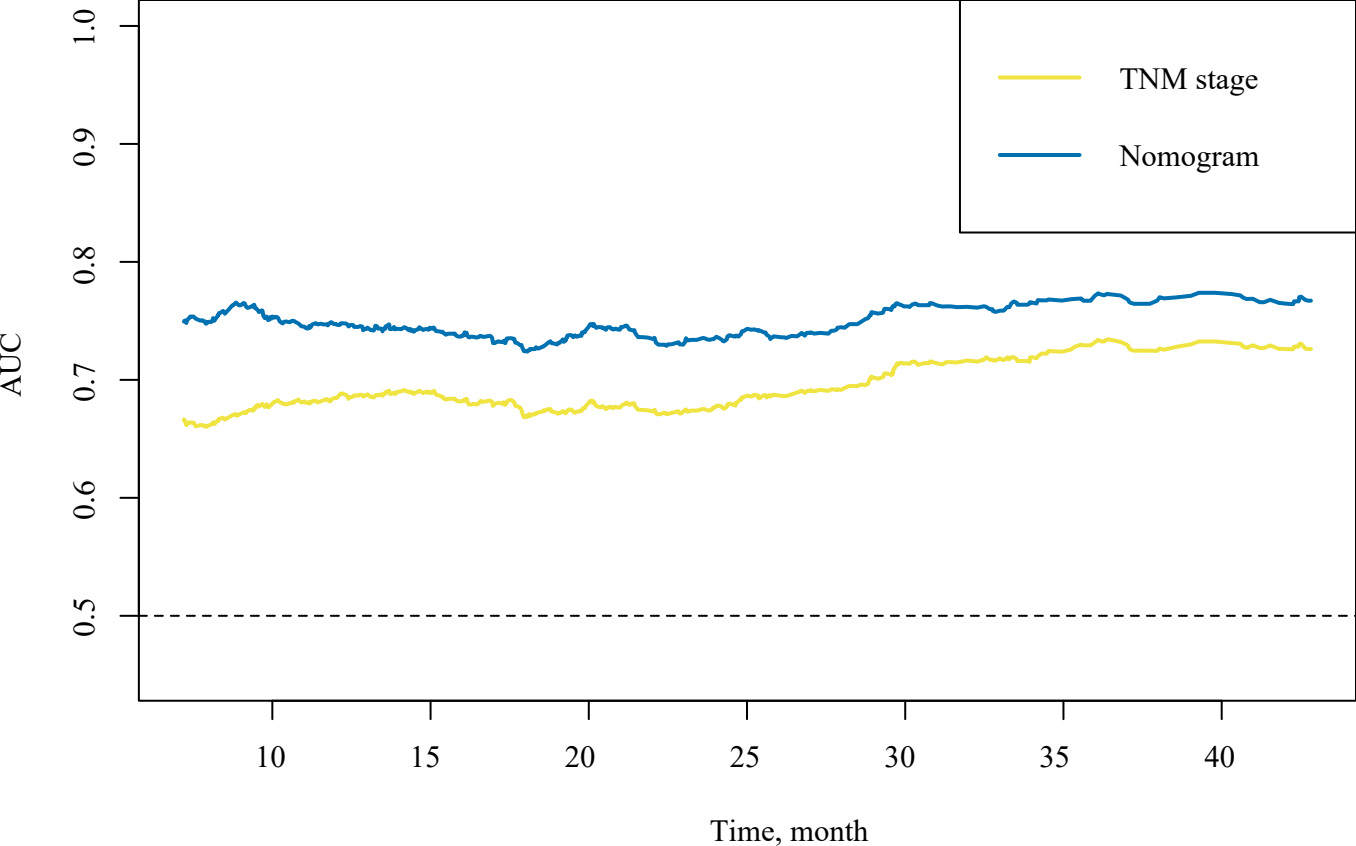

Supplement: Supplementary Figure 6 — Area Under the Curves (AUCs) of time-dependent receiver operating characteristic curves (ROCs) generated based on the TNM stage and the nomogram for patients with non-small cell lung cancer (NSCLC). [file Image_6.PDF]

**(A)**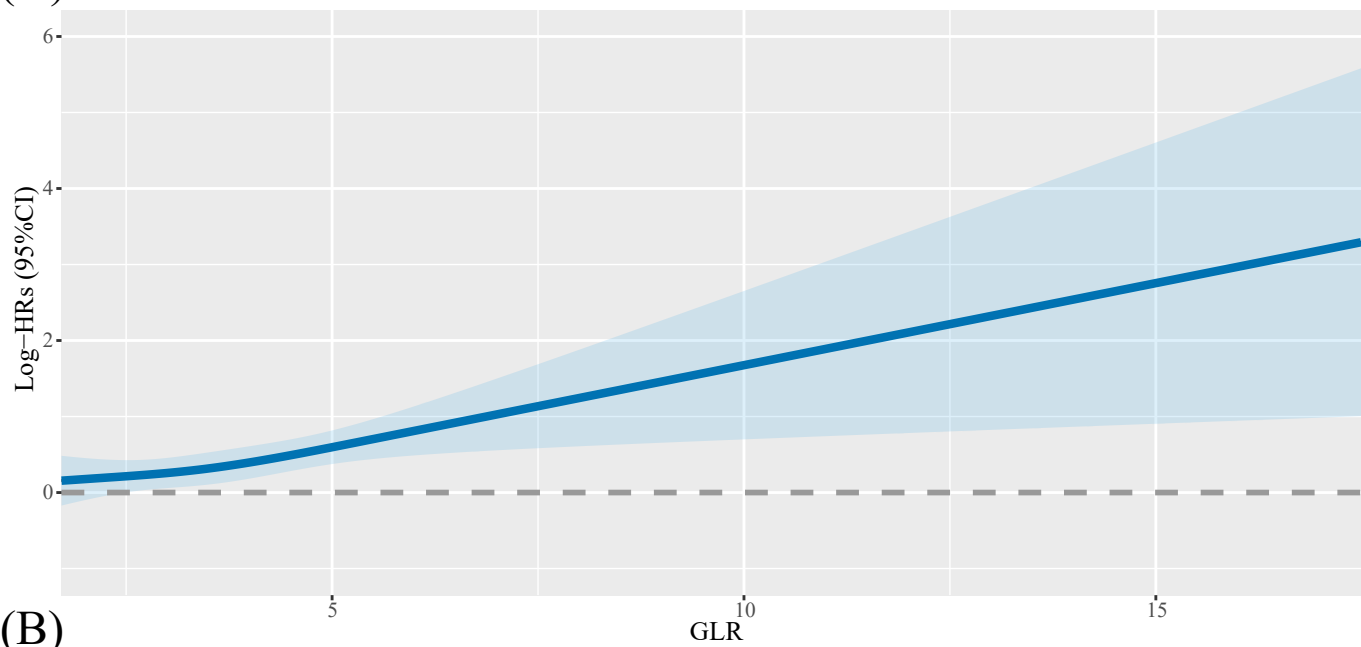**(B)**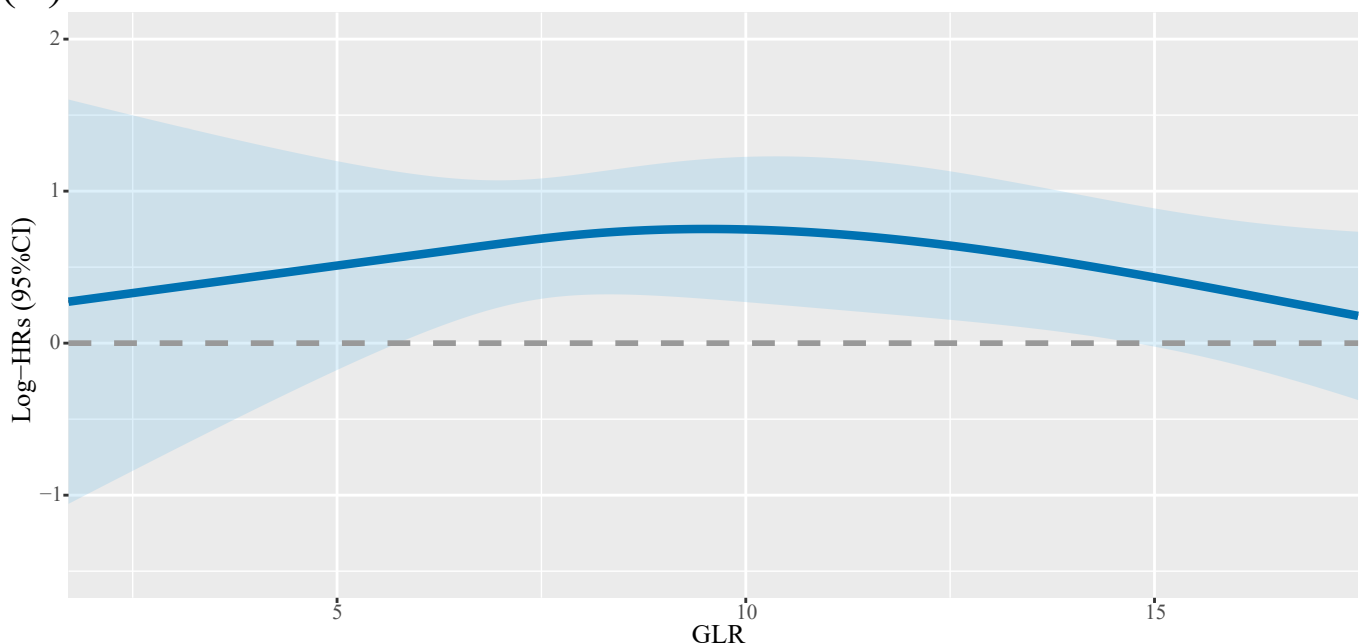

Supplement: Supplementary Figure 7 — Area Under the Curves (AUCs) of TNM stage and the nomogram in patients with non-small cell lung cancer (NSCLC). [file Image_7.PDF]

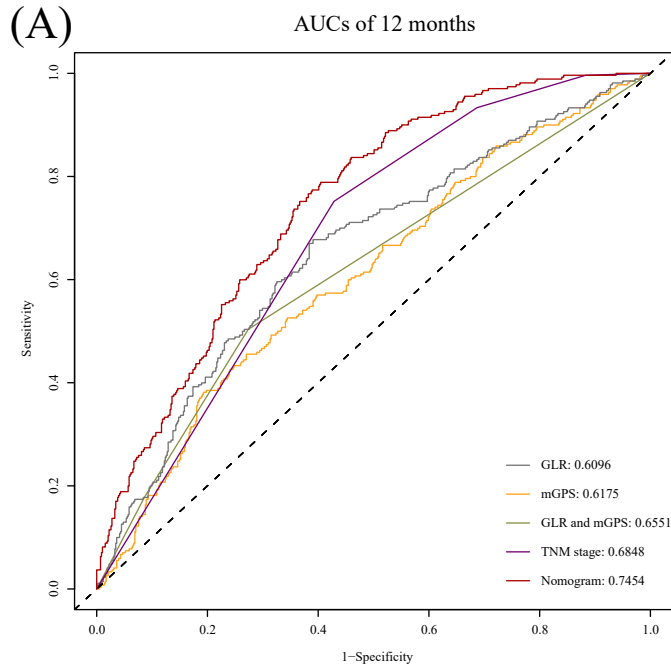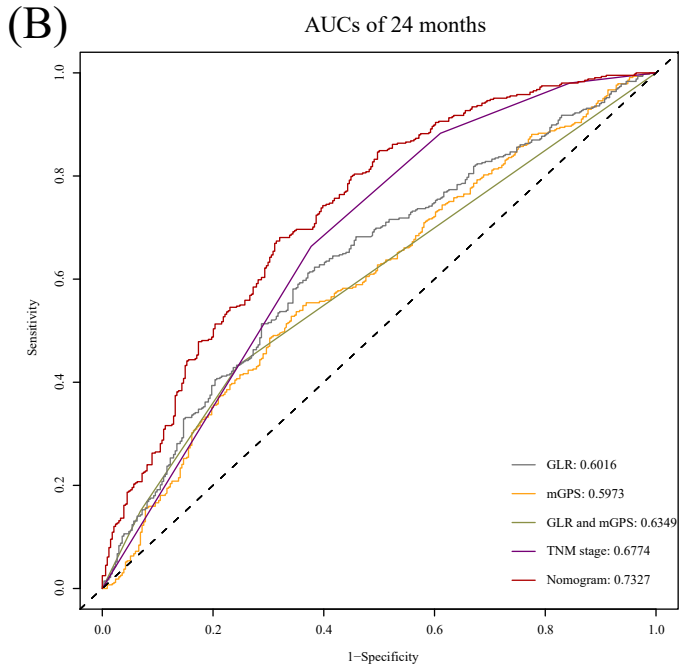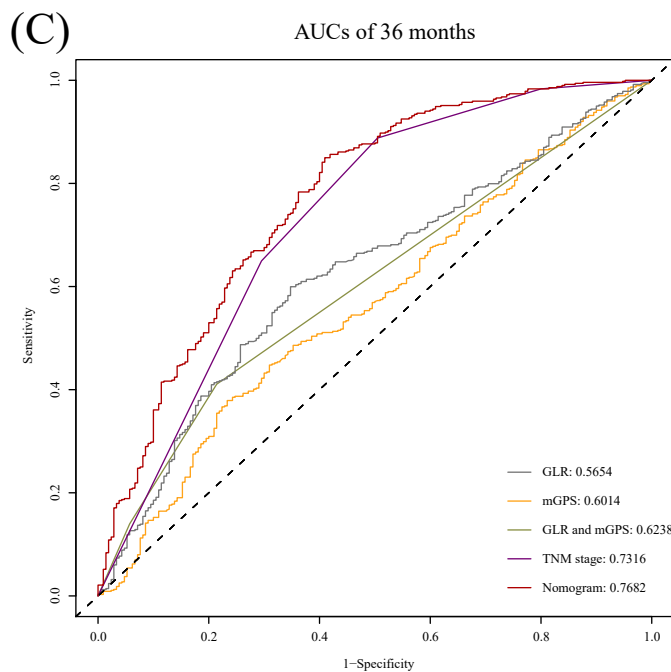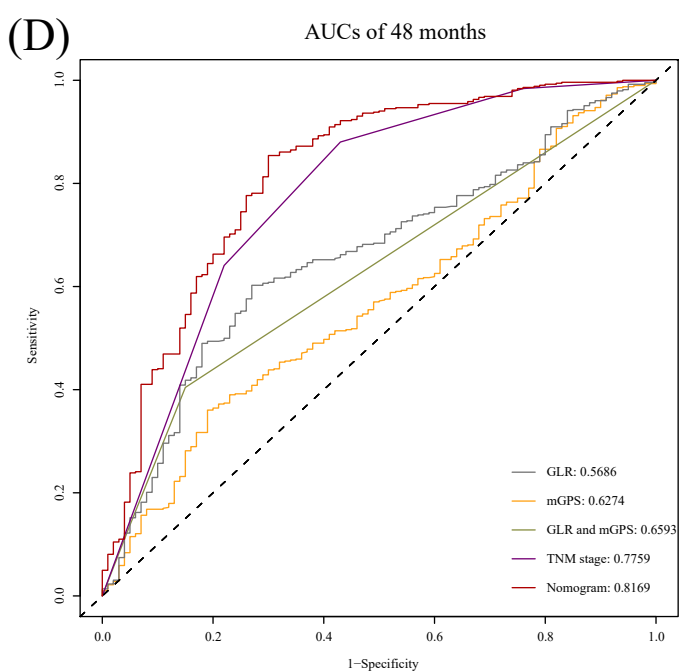

Supplement: Supplementary Figure 8 — Associations between blood glucose to lymphocyte ratio (GLR <6.25 or ≥ 6.25) and OS in patients with non-small cell lung cancer (NSCLC). The models were adjusted for sex, age, BMI, tea drinking status, TNM stage and modified Glasgow prognostic score (mGPS). [file Image_8.PDF]
