# Supplementary material for: Prognostic Roles of Glucose to Lymphocyte Ratio and Modified Glasgow Prognosis Score in Patients With Non-small Cell Lung Cancer
Source: Front Nutr. 2022 May 10;9:871301. doi: 10.3389/fnut.2022.871301 (PMC9127733; doi:10.3389/fnut.2022.871301)
Supplement: Supplementary Table 1 — Associations between GLR and OS in patients with NSCLC. GLR, blood glucose to lymphocyte ratio; OS, overall survival; NSCLC, non-small cell lung cancer; HR, hazard ratio; CIs, confidence intervals. a Models were adjusted for sex, age, body mass index, tea drinking status, TNM stage and mGPS. [file Table_1.DOCX]

**Supplemental table 1 Associations between GLR and OS in patients with NSCLC.**

| Variables | Patients (n) | Crude HR (95% CIs) | *P* value | Adjusted HR (95% CIs) | *P* value |
| --- | --- | --- | --- | --- | --- |
| GLR *^a^* |  |  |  |  |  |
| <6.25 | 727 | 1.218 (1.119, 1.326) | <0.001 | 1.171 (1.074, 1.276) | <0.001 |
| ≥6.25 | 135 | 0.973 (0.926, 1.023) | 0.289 | 0.958 (0.907, 1.011) | 0.117 |

Notes:

Abbreviations: GLR: blood glucose to lymphocyte ratio, OS: overall survival, NSCLC: non-small cell lung cancer, HR: hazard ratio, CIs: confidence intervals.

***^a^*** Models were adjusted for sex, age, body mass index, tea drinking status, TNM stage and mGPS.
